# Chemical Composition of the Essential Oils of *Cyperus rotundus* L. from South Africa

**DOI:** 10.3390/molecules14082909

**Published:** 2009-08-06

**Authors:** Oladipupo A. Lawal, Adebola O. Oyedeji

**Affiliations:** Department of Chemistry, University of Zululand, KwaDlangezwa 3886, South Africa

**Keywords:** *Cyperus rotundus*, Cyperaceae, essential oil composition, α-cyperone, β-pinene α-pinene, α-selinene, caryophyllene oxide

## Abstract

The essential oils from the rhizomes of *Cyperus rotundus* L. collected from two different locations (Empangeni-A and KwaDlangezwa-B; both in the Kwa-Zulu Natal Province of South Africa) were obtained by hydrodistillation and analyzed by capillary GC and GC/MS. Forty-one and 43 components were identified, representing 89.9% and 92.0% of sample A and sample B, respectively. α-Cyperone (11.0%), myrtenol (7.9%), caryophyllene oxide (5.4%) and β-pinene (5.3%) were major compounds in the oil of sample A. The main constituents of the oil of sample B were β-pinene (11.3%), α-pinene (10.8%), α- cyperone (7.9%), myrtenol (7.1%) and α-selinene (6.6%).

## Introduction

*Cyperus rotundus* L., (family Cyperaceae), also known as purple nutsedge or nutgrass, is a common perennial weed with slender, scaly creeping rhizomes, bulbous at the base and arising singly from the tubers which are about 1-3 cm long. The tubers are externally blackish in colour and reddish white inside, with a characteristic odour. The stems grow to about 25 cm tall and the leaves are linear, dark green and grooved on the upper surface. Inflorescences are small, with 2-4 bracts, consisting of tiny flowers with a red-brown husk. The nut is three-angled, oblong-ovate, yellow in colour and black when ripe. *C. rotundus* is indigenous to India, but are now found in tropical, subtropical and temperate regions [1,2].

*Cyperus rotundus* is a multipurpose plant, widely used in traditional medicine around the world to treat stomach aliments, wounds, boils and blisters [3,4,5,6]. A number of pharmacological and biological activities including anti-*Candida*, anti-inflammatory, antidiabetic, antidiarrhoeal, cytoprotective, antimutagenic, antimicrobial, antibacterial, antioxidant, cytotoxic and apoptotic, anti-pyretic and analgesic activities have been reported for this plant [7,8,9,10,11,12,13,14,15,16,17]. Previous phytochemical studies on *C. rotundus* revealed the presence of alkaloids, flavonoids, tannins, starch, glycosides and furochromones, and many novel sesquiterpenoids [9,18,19,20,21,22,23,24,25,26,27,28,29].

The chemical composition of the volatile oils of *C. rotundus* has been extensively studied and four chemotypes (H-, K-, M- O-types), of the essential oils from different parts of Asia have been reported [14,30,31,32,33,34,35]. The H-type from Japan was found to contain α-cyperone (36.6%), β-selinene (18.5%), cyperol (7.4%) and caryophyllene (6.2%). The M-type from China, Hong Kong, Japan, Taiwan and Vietnam had α-cyperone (30.7%), cyperotundone (19.4%), β-selinene (17.8%), cyperene (7.2%) and cyperol (5.6%). The O-type from Japan, Taiwan, Thailand, Hawaii and the Philippines was characterized by cyperene (30.8%), cyperotundone (13.1%) and β-elemene (5.2%). In addition, the Hawaiian O-type had cyperotundone (25.0%) and cyperene (20.7%) as the major compounds. Finally, the K-type, also from Hawaii, was dominated by cyperene (28.7%), cyperotundone (8.8%), patchoulenyl acetate (8.0%) and sugeonyl acetate (6.9%) [30,31].

The rhizome oils of this plant from different countries also showed compositional differences, suggesting the existence of phytochemical varieties. Cyperene (19.2-30.9%) and α-cyperone (4.5-25.2%) were the most abundant constituents of the oils of Nigerian and Tunisian species, but the concentrations of other main components varied [32,33]. The Brazilian species was found to contain α-cyperone (22.8%) and cyperotundone (12.1%) as the main compounds of the oil [34]. The rhizome oils of *C. rotundus* from India were reported to have α-copaene (11.4-12.1%), cyperene (8.4-11.7%), valerenal (8.7-9.8%), caryophyllene oxide (7.8-9.7%) and *trans*-pinocarveol (5.2-7.4%), some of which were absent in the species from other countries [35]. Sonwa and Koenig [36] investigated the essential oil of *C. rotundus* from Germany, and found the oil to be dominated by cyprotene, α-copaene, cyperene, α-selinene, rotundene, cadalene and nootkatene, among others.

As part of our on-going research on the chemical composition of the essential oils of South African medicinal and aromatic plants from the family Cyperaceae, the present investigation reports on the essential oils from the rhizomes of *Cyperus rotundus* found in South Africa.

## Results and Discussion

Hydrodistillation of the fresh rhizomes of *C. rotundus* collected from the two different locations yielded 0.20% and 0.16% pale yellowish oils for Empangeni (sample A) and KwaDlangezwa (sample B), respectively. The compositions of the two oils are displayed in Table 1, where constituents are listed in order of their elution on the (DB-5) column. A total 58 components were detected, 41 and 43 of which were identified, accounting for 88.9% and 92.0% of the oil of Empangeni and KwaDlangezwa samples, respectively. The oil of the Empangeni sample was characterized by larger amounts of sesquiterpenes (59.8%) than monoterperenes (29.1%), while the KwaDlangezwa oil sample had a relatively similar content of sesquiterpenes (45.9%) and monoterperenes (46.1%). The sesquiterpenic composition of the oil of Empangeni is dominated by α-cyperone (11.0%), caryophyllene oxide (5.4%) and β-selinene (5.1%), and the compounds: myrtenol (7.9%), β-pinene (5.3%) and *trans*-pinocarveol (4.0%) were the major representative of monoterpenoids. In the oil of KwaDlangezwa, β-pinene (11.3%), α-pinene (10.8%), α-cyperone (7.9%), myrtenol (7.1%), α-selinene (6.6%), limonene (5.7%) and β-selinene (4.6%) were the main constituents. Although the compositions of the oil from each location varied, certain similarities were evident. The composition profile of the oil of Empangeni sample shows that it is richer in sesquiterpenes and is therefore, similar to other reported essential oils compositions of *C. rotundus* from different countries [14,30,31,32,33,34,35]. However, the chemical pattern of the oil of KwaDlangezwa sample is completely different from the sesquiterpenoid dominating types.

**Table 1:** Percentage composition of *C. rotundus* essential oils from two different locations.

Compounds	RIa	Sample A	Sample B	Method of Identification
α-pinene	936	3.0	10.8	MS, RI
camphene	951	t	1.5	MS, RI
β-pinene	979	5.3	11.3	MS, RI
myrcene	990	0.5	-	MS, RI
α-phellandrene	1002	t	-	MS, RI
bicyclo [3.2.0] hept-6-ene *	1014	-	0.3	RI
*p*-cymene	1026	1.7	0.6	MS, RI
limonene	1030	2.0	5.7	MS, RI
1,8-cineole	1033	t	-	MS, RI
terpinolene	1082	0.6	-	MS, RI
perillene	1096	-	0.3	MS, RI
3,3,5-trimethyl cyclohexene	1118	-	0.2	RI
fenchol	1121	-	0.2	MS, RI
*trans*-pinocarveol	1142	4.0	4.0	MS, RI
camphene hydrate	1147	-	0.4	MS, RI
pinocarvone	1158	2.2	0.4	MS, RI
*p*-mentha-1, 5-diene-8-ol	1161	0.4	-	MS, RI
borneol	1167	-	0.3	MS, RI
terpinen-4-ol	1178	0.9	1.0	MS, RI
myrtenol	1201	7.9	7.1	MS, RI
verbenone	1209	0.6	1.1	MS, RI
*trans*-carveol	1226	t	0.4	MS, RI
cuminaldehyde	1251	t	0.1	MS, RI
carvone	1257	-	0.2	MS, RI
α-copaene	1379	-	0.5	MS, RI
β-elemene	1383	0.8	0.5	MS, RI
cyperene	1397	1.6	2.6	MS, RI
β-caryophyllene	1426	0.8	0.6	MS, RI
α-gurjunene	1431	-	0.3	MS, RI
α-humulene	1452	0.4	0.2	MS, RI
*allo*-aromadendrene	1468	1.2	0.8	MS, RI
eudesma-2,4,11-triene	1476	2.1	-	MS, RI
β-selinene	1484	5.1	4.6	MS, RI
α-selinene	1491	2.7	6.6	MS, RI
germacrene B	1546	-	2.1	MS, RI
spathulenol	1572	0.3	-	MS, RI
caryophyllene oxide	1584	5.4	2.6	MS, RI
(2R,5E)-caryophyll-5-en-12-al	1593	1.0	-	MS, RI
humulene epoxide II	1601	2.7	1.6	MS, RI
oplopenone	1608	3.4	-	RI
globulol	1623	-	0.9	MS, RI
patchenol **	1628	3.9	0.9	RI
2-cyclopropylthiophene	1631	-	2.5	RI
caryophylla-3,8(13)-dien-5-β-ol	1641	4.2	2.4	RI
vulgarol B	1642	3.8	1.8	RI
caryophylla-3,8(13)-dien-5-α-ol	1649	2.1	-	RI
caryophyllenol 11	1661	4.8	0.9	RI
aromadendrene epoxide	1743	-	2.7	RI
aristolone	1752	2.5	1.6	MS, RI
α-cyperone	1771	11.0	7.9	MS, RI
oxo-α-ylangene	1779	-	1.9	MS, RI
M^+^ 218 (C_15_H_22_O) **	1783	1.4	-	RI
M^+^ 218 (C_15_H_22_O) **	1796	1.7	1.1	RI
solavetivone	1816	t	-	MS, RI
nootkatone	1820	-	0.2	MS, RI
hexadecanoic acid	1942	t	-	MS, RI
phytol	2096	t	-	MS, RI
Monoterpene hydrocarbons		13.1	30.4	
Oxygenated monoterpenes		16.0	15.5	
Sesquiterpene hydrocarbons		14.7	18.2	
Oxygenated sesquiterpenes		45.1	27.9	
Total identified		88.9	92.0	
unidentified		3.1	1.1	

RI^a^ = Retention Indices relative to C_9_-C_24_ n-alkanes on the DB-5 column; t = trace amount (≤0.05%); Empangeni = Sample A; KwaDlangezwa = Sample B; * Correct isomer not identified; ** An alcohol of methanoazulene; *** tentatively identified: RI^a^ = 1783: [M^+.^] 218(100), 41(98), 91(93), 55(70), 79(69), 105(68), 67(63), 203(60); RI^a^ = 1796: [M^+.^] 95(100), 69(52), 179(50), 41(42), 133(38), 79(28), 107(25), 218(22).

In all the previous reports on the chemical composition of essential oils of *C. rotundus* from around the world, α-cyperone, cyperene, cyperotundone and β-selinene were found to be the major compounds identified in higher concentrations, along with other constituents such as, α-copaene, valerenal, caryophyllene oxide, patchoulenyl acetate and sugeonyl acetate (Table 2). In addition, some reports have had the occurrence of α-pinene, β-pinene, limonene and 1,8-cineole as minor components of the essential oils of *C. rotundus* [14,15]. However, in our results (Table 1 and Table 2), cyperene, α-cyperone and β-selinene were present only in small concentrations, while cyperotundone, valerenal, petchoulenyl and sugeonyl acetates were not detected at all in our oil samples. Interestingly, the major constituents of the KwaDlangezwa oil sample, α-pinene (10.8%) and β-pinene (11.3%) have not been previously reported in dominant quantities among the major compounds from the oils of *C. rotundus* (Table 2).

**Table 2:** Comparison of major constituents of *C. rotundus* rhizomes oils from different countries.

Oil percentage composition										
Constituents	1	2	3	4	5	6	7	8	9	10
α-pinene	-	-	-	-	-	-	-	-	-	(3.0-10.8)
β-pinene	-	-	-	-	-	-	-	-	-	(5.3-11.3)
α-copaene	-	-	-	-	-	-	(11.4-12.1)	-	-	-
cyperene	-	30.8	7.2	28.7	20.7	-	(8.4-11.1)	19.2	(20.4-30.9)	(1.6-2.6)
β-selinene	18.3	-	17.8	-	-	-	-	-	-	(4.6-5.1)
cyperotundone	-	13.1	19.4	8.8	25.0	12.1	--	-	8.8	-
α-cyperone	38.6	-	30.7	-	-	22.8	-	17.7	(4.5 – 25.2)	(7.9-11.0)
valerenal	-	-	-	-	-	-	(8.7-9.8)	-	-	-
caryophyllene oxide	-	-	-	-	-	-	(7.8-9.7)	-	-	(2.6-5.4)
petchoulenyl acetate	-	-	-	8.0	-	-	-	-	-	-
sugeonyl acetate	-	-	-	8.9	-	-	-	-	-	-

1 = H-type; 2 = O-type; 3 = M-type; 4 = K-type; 5 = Hawaiian O-type (Asia countries) [31,32]; 6 = Brazil [35]; 7 = India [36]; 8 = Nigeria [33]; 9 = Tunisia [14,34], 10 = South Africa (present study).

Comparing the present results with those previously reported in the literature on the essential oil compositions of *C. rotundus* from different countries [14,30,31,32,33,34,35], it is apparent that, there are many differences regarding the major constituents of the oils of *C. rotundus,* which further suggests the existence of more chemical diversity within the *C. rotundus* species [33]. However, cyperene and α-cyperone are the two major compounds similar in the essential oils of *C. rotundus* from Africa (Nigeria, Tunisia and South Africa). The observed compositional difference between *C. rotundus* found in South Africa and the rest of the world could be due to climactic and environmental conditions, chemotypes, nutritional status of the plants, and other factors, which can influence essential oil composition [14,30,31,37,38,39,40,41]. In conclusion, the essential oils of *C. rotundus* from South Africa can be related to the M-type due to the presence of cyperene, β-selinene and α-cyperone.

## Experimental

### Plant material

Fresh plant materials of *C. rotundus* growing wild on the campus of University of Zululand, KwaDlangezwa (Sample B) and along Empangeni road (Sample A) in the KwaZulu-Natal Province of South Africa were randomly collected in March, 2007. Mrs. N.R Ntuli of Department of Botany, University of Zululand, identified the plant materials. Voucher specimens [Lawal, OA 05 and 06 (ZULU)] were deposited at the University of Zululand Herbarium.

### Oil isolation

Finely chopped fresh rhizomes of each sample (500 g) were separately subjected to hydrodistillation in a Clevenger-type apparatus for 4 h in accordance with the British Pharmacopoeia specification [42]. The sample was added to distilled deionized water (1.5 L) in a 2-5 L round-bottomed flask and heated to boiling, after which the essential oil was evaporated together with water vapour and finally collected in a condenser. The upper phase that contained the essential oil was separated from the lower one and the distillate isolated was preserved in a sealed sample tube and stored under refrigeration until analysis [43,44].

### GC analyses

GC analysis was carried out on a Hewlett Packard HP 6820 Gas Chromatograph equipped with a FID detector and DB-5 column (60 m x 0.25 mm id), film thickness was 0.25 µm and the split ratio was 1:25. The oven temperature was programmed from 50 °C (after 2 min) to 240 °C at 5 °C/min and the final temperature was held for 10 min. Injection and detector temperatures were 200 °C and 240 °C, respectively. Hydrogen was the carrier gas. An aliquot (0.5 µL of the diluted oil) was injected into the GC. Peaks were measured by electronic integration. A homologous series of *n*-alkanes were run under the same conditions for determination of retention indices.

### GC-M S analyses

GC-MS analyses of the oils were performed on a Hewlett Packard HP 6890 Gas Chromatography interfaced with Hewlett Packard 5973 mass spectrometer system equipped with a DB-5 capillary column (30 m x 0.25 mm id, film thickness 0.25 µm). The oven temperature was programmed from 70- 240 °C at the rate of 5 °C/min. The ion source was set at 240 °C and electron ionization at 70 eV. Helium was used as the carrier gas at a flow rate of 1 mL/min. Scanning range was 35 to 425 amu. 1.0 µL of diluted oil in hexane was injected into the GC/MS.

### Identification of components

The components of the oils were identified base on the comparison of their retention indices and mass spectra with those standards, Wiley 275 library mass spectra databased of the GC/MS system and published data [45,46,47].

## References

[1] Pooley E. (1998). A Field Guide to Wild Flowers in KwaZulu-Natal and Eastern Region.

[2] Gordon-Gray K.D. (1995). Cyperaceae in Natal.

[3] Oliver-Bever B. (1986). Medicinal Plants in Tropical West Africa.

[4] Puratuchikody A., Nithya D.C., Nagalakshmi G. (2006). Wound Healing Activity of *Cyperus rotundus* Linn.. Indian J. Pharm. Sci..

[5] Joshi A.R., Joshi K. (2000). Indigenous knowledge and uses of medicinal plants by local communities of the Kali Gandaki Watershed Area, Nepal. J. Ethnopharmacol..

[6] El-Kamali H.H., El-Khalifa K.F. (1999). Folk medicinal plants of riverside forests of the Southern Blue Nile district, Sudan. Fitoterapia.

[7] Durate M.C.T., Figueira G.M., Sartoratto A., Rehder V.L.G., Delarmelina C. (2005). Anti-Candida activity of Brazilian medicinal plant. J. Ethnopharmacol..

[8] Sundaram M.S., Sivakumar T., Balamurugan G. (2008). Anti-inflammatory effect of *Cyperus rotundus* Linn. Leaves on acute and subacute inflammation in experimental rat models. Biomedicine.

[9] Raut N.A., Gaikwad N.J. (2006). Antidiabetic activity of hydro-ethanolic extract of *Cyperus rotundus* in alloxan induced diabetes in rats. Fitoterapia.

[10] Uddin S.J., Mondal K., Shilpi J.A., Rahman M.T. (2006). Antidiarrhoeal activity of *Cyperus rotundus*. Fitoterapia.

[11] Kilani S., Ben Ammar R., Bouhlel I., Abdelwahed A., Hayder N., Mahmoud A., Ghedira K., Chekir-Ghedira L. (2005). Investigation of extracts from (Tunisian) *Cyperus rotundus* as antimutagens and radical scavengers. Environ. Toxicol. Pharmacol..

[12] Zhu M., Luk H.H., Fung H.S., Luk C.T. (1997). Cytoprotective effects of *Cyperus rotundus* against ethanol induced gastric ulceration in rats. Phytother. Res..

[13] Kilani S., Bouhlel I., Ben Ammar R., Ben Sghair M., Skandrani I., Boubaker J., Mahmoud A., Dijoux-Franca M.G., Ghedira K., Chekir-Ghedira L. (2007). Chemical investigation of different extracts and essential oil from the tubers of (Tunisian) *Cyperus rotundus*. Correlation with their antiradical and antimutagenic properties. Ann. Microbiol..

[14] Kilani S., Ledauphin J., Bouhlel I., Ben Sghaier M., Boubaker J., Skandrani I., Mosrati R., Ghedira K., Barillier D., Chekir-Ghedira L. (2008). Comparative study of *Cyperus rotundus* essential oil by a modified GC/MS analysis method. Evaluation of its antioxidant, cytotoxic, and apoptotic effects. Chem. Biodivers..

[15] Dhillon R.S., Singh S., Kundra S., Basra A.S. (1993). Studies on the chemical composition and biological activity of essential oil from *Cyperus rotundus* Linn.. Plant Growth Regul..

[16] Pal D.K., Dutta S. (2006). Evaluation of the Antioxidant activity of the roots and Rhizomes of *Cyperus rotundus* L.. Indian J. Pharm. Sci..

[17] Kilani S., Ben Sghaier M., Limem I., Bouhlel I., Boubaker J., Bhouri W., Skandrani I., Neffatti A., Ben Ammar R., Dijoux-Franca M.G., Ghedira K., Chekir-Ghedira L. (2008). *In vitro* evaluation of antibacterial, antioxidant, cytotoxic and apoptotic activities of the tubers infusion and extracts of *Cyperus rotundus*. Bioresour. Technol..

[18] EL-Habashy I., Mansour R.M.A., Zahran M.A., EL-Hadid M.M., Saleh N.A. (1989). Leaf flavonoids of *Cyperus* species in Egypt. Bio. Syst. Ecol..

[19] Harborne JB., Williams C.A., Wilson K.L. (1982). Flavonoids in leaves and inflorescences of Australian *Cyperus* species. Phytochemistry.

[20] Umerie S.C., Ezeuzo H.O. (2000). Physicochemical characterization and utilization of *Cyperus rotundus* starch. Bioresour. Technol..

[21] Sayed H.M., Mohamed M.H., Farag S.F., Mohamed G.A., Proksch P. (2007). A new steroid glycoside and furochromones from *Cyperus rotundus* L.. Nat. Prod. Res..

[22] Kapadia V.H., Naik V.G., Wadia M.S., Dev S. (1967). Sesquiterpenoids from Essential oil of *Cyperus rotundus*. Tetrahedron Lett..

[23] Trivedi B., Motl O., Herout V., Sorm F. (1984). Composition of the oil from *Cyperus rotundus*: Structure of patchoulenone. Coil. Czech. Chem. Commun..

[24] Thebtaranonth C., Thebtaranonth Y., Wanauppaphamkul C., Yuthavong Y. (1995). Antimalarial sesquiterpenes from tubers of *Cyperus rotundus*. Structure of 10, 12-peroxycalamenene, Asesquiterpenes endoperoxide. Phytochemistry.

[25] Ohira S., Hasegawa T., Hyashi K.I., Hoshino T., Takaoka D., Nozaki H. (1998). Sesquiterpenoids from *Cyperus rotundus*. Phytochemistry.

[26] Jeong S.J., Miyamoto T., Inagaki M., Kim Y.C., Higuchi R. (2000). Rotundines A-C, three novel sesquiterpene alkaloids from *Cyperus rotundus*. J. Nat. Prod..

[27] Kilani S., Ben Ammar R., Bouhlel I., Abdelwahed A., Hayder N., Mahmoud A., Ghedir K., Chekir-Ghedira L. (2005). Investigation of extracts from (Tunisian) *Cyperus rotundus* as antimutagens and radical scavengers. Environ. Toxicol. Pharmacol..

[28] Morimoto M., Komai K. (2005). Plant growth inhibitors: Patchoulane-type sesquiterpenes from *Cyperus rotundus* L.. Weed Biol. Manag..

[29] Xu Y., Zhang H., Yu C., Lu Y., Chang Y., Zou Z. (2008). Norcyperone, a Novel Skeleton Norsesquiterpene from *Cyperus rotundus* L.. Molecules.

[30] Komai K., Tang C. (1989). A Chemotype of *Cyperus rotundus* in Hawaii. Phytochemistry.

[31] Komai K., Shimizu M., Tang C.T., Tsutsui H. (1994). Sesquiterpenoids of *Cyperus bulbosus*, *Cyperus tuberosus* and *Cyperus rotundus*. Mem. Fac. Agr. Kinki Univ..

[32] Ekundayo O., Oderinde R., Ogundeyin. M., Biskup E.S. (1991). Essential oil constituents of *Cyperus tuberosus* Rottb. Rhizomes. Flav. Fragr. J..

[33] Kilani S., Abdelwahed A., Chraief I., Ben Ammar R., Hayder N., Hammami M., Ghedira K., Chekir-Ghedira L. (2005). Chemical composition, antibacterial and antimutagenic activities of essential oil from (Tunisian) *Cyperus rotundus*. J. Essent. Oil Res..

[34] Zoghbi M.D.G.B., Andrade E.H.A., Carreira L.M.M., Rocha E.A.S. (2008). Comparison of the main components of the essential oils of "priprioca": *Cyperus articulatus* var. *articulatus* L., *C. articulatus* var. *nodosus* L., *C. prolixus* Kunth and *C. rotundus* L.. J. Essent. Oil Res..

[35] Jirovetz L., Wobus A., Buchbauer G., Shafi M.P., Thampi P.T. (2004). Comparative analysis of the essential oil and SPME-headspace aroma compounds of *Cyperus rotundus* L. roots/tubers from South-India using GC, GC-MS and olfactometry. J. Essent. Oil-Bearing Plants.

[36] Sonwa M.M., Koenig W.A. (2001). Chemical study of essential oil *Cyperus rotundus*. Phytochemistry.

[37] Loziene K., Venskutonis P.R. (2005). Influence of environmental and genetic factors on the stability of essential oil composition of *Thymus pulegioides*. Bio. Syst. Ecol..

[38] Clark R.J., Menary R.C. (1980). Environmental effects on peppermint (*Mentha piperita* L.). II. Effects of temperature on photosynthesis, photorespiration and dark respiration in peppermint with reference to oil composition. Aust. J. Plant Physiol..

[39] Boira H., Blanquer A. (1998). Environmental factors affecting chemical variability of essential oils in *Thymus piperella* L.. Bio. Syst. Ecol..

[40] Burbott A.J., Loomis W.D. (1967). Effects of light and temperature on the monoterpenes of peppermint. Plant Physiol..

[41] Lahlou M. (2004). Methods to study the phytochemistry and bioactivity of essential oils. Phytother. Res..

[42] (1988). British Pharmacopoeia Part II.

[43] Oyedeji O.A., Ekundayo O., Koenig W.A. (1999). Constituents of the essential oils from the leaves of *Lenontis nepetaefolia* (L.) Ait. F.. J. Essent. Oil Res..

[44] Oyedeji O.A., Ekundayo O., Olawore O.N., Koenig W.A. (2000). Essential oil composition of two varieties of *Eucalyptus camaldulensis* from Nigeria. J. Essent. Oil Res..

[45] Adams R.P. (1989). Identification of Essential Oil Components by Ion Trap Mass Spectroscopy.

[46] Joulain D., Koenig. W.A. (1998). The Atlas of Spectral Data of Sesquiterpenes Hydrocarbons.

[47] (1999). ESO 2000. The complete database of essential oils.

